# Local Positioning System Analysis of Physical Demands during Official Matches in the Spanish Futsal League

**DOI:** 10.3390/s20174860

**Published:** 2020-08-28

**Authors:** Carlos Serrano, Jose Luis Felipe, Jorge Garcia-Unanue, Enrique Ibañez, Enrique Hernando, Leonor Gallardo, Javier Sanchez-Sanchez

**Affiliations:** 1 IGOID Research Group, Physical Activity and Sport Sciences Department, University of Castilla-La Mancha, 45071 Toledo, Spain; carlos.serrano.90@hotmail.com (C.S.); jorge.garciaunanue@uclm.es (J.G.-U.); Enrique.Hernando@uclm.es (E.H.); leonor.gallardo@uclm.es (L.G.); 2 School of Sport Sciences, Universidad Europea de Madrid, 28670 Villaviciosa de Odón (Madrid), Spain; javier.sanchez2@universidadeuropea.es; 3 Movistar Inter Fútbol Sala, 28850 Torrejón de Ardoz (Madrid), Spain; enriqueibanezramos@outlook.es

**Keywords:** team sport, match monitoring, physical performance, indoor tracking system

## Abstract

The aim of this study was to analyze the influence of the match half and the playing position on physical requirements in the Spanish Professional Futsal League players during official games. The external load from distance, speed, acceleration and deceleration variables were obtained from fourteen elite futsal players during 10 official matches of the 2019–2020 season using a Local Positioning System with ultra-wideband technology installed on the futsal pitch. The results revealed similar results from physical requirements between first and second half (*p* > 0.05). Wingers demonstrated greater high-speed running distance (+4.04 m·min^−1^; CI95%: 0.35 to 7.72; ES: 0.87) than pivots (*p* > 0.05). There were a high number of accelerations (7.42–9.41 *n*·min^−1^) and decelerations (7.37–9.12 *n*·min^−1^) per minute in all player positions. The principal finding of the current manuscript did not evidence differences in the physical performance of players between the first and second half. The physical requirements varied among pivots and wingers regarding high-intensity actions. These outcomes add new contributions to the understanding of futsal physical demands.

## 1. Introduction

Futsal is a team sport whose principal characteristics are high-intensity effort and great technical and tactical exigences [1]. Futsal players need to have, or develop, a great capacity for intermittent endurance, repeated sprint ability, explosive strength of lower limbs and agility [2]. Furthermore, physical performance could depend on contextual variables that exist during matches [3,4]. Identification and quantification of key performance aspects is important because it potentially affects various aspects of the game [5] and helps in developing team-based training and analyzing individual athletic performance [6].

During the last few years, wearable technology such as global positioning systems (GPS) or other tracking sensors have begun to provide more valid, reliable and time-efficient measures that quantify and track team sport-specific demands during play, particularly in outdoor sports [7,8]. Recently, the progress in this kind of technology has made it possible to develop a Local Positioning System (LPS) with ultra-wideband technology (UWB) [9,10] to track indoor team sport requirements [11], making it possible to track time–motion analysis and physical demands in basketball [12], handball [13], and futsal [14]. This system is fixed and is not affected by environmental conditions [15], and the validity and accuracy of this type of LPS has been demonstrated [10].

Studies have indicated that futsal players may have a high level of aerobic and anerobic ability as far as physical demands are concerned [2,16] as the match time is stopped for various events that occur in a game, thus the real duration of a match might exceed the established official time of 40 min [17]. Therefore, the official duration of a match may vary from 75 to 90 min [17]. Moreover, players perform a low-intensity effort every 14 s, a medium-intensity effort every 37 s, a high-intensity effort every 43 s, a maximum-intensity effort every 56 s, with 8.6 activities per minute of match play and change locomotor activities every 3.3 s [2,5]. Thus, repeat sprint ability is absolutely essential because of the numerous sprint actions followed by short periods of rest [18]. In turn, this sport requires players to possess power, strength, agility and coordination [2], in order for them to perform several actions, such as changes of direction, accelerations and decelerations [6].

It has been observed in different professional leagues that players are able to run a total distance of between 3000 and 4000 m [2,14]. Professional futsal players cover between 10.3% and 13.7% of their total distance at high-intensity, and sprinting between 8.9% and 10.1% [2,19]. It is likely that this total distance run varies according to the number of players involved in a match owing to the unlimited substitutions rule in futsal [19]. Preceding researches have reported a total distance per minute covered during a game between 108 and 232 m [2,14]. Additionally, the repeat-sprint-sequence more predominantly is 2 or 3 sprints with a recovery interval of up to 15 s between them [18]. Moreover, these futsal player participants seem to be affected by their position on the field, tactical disposition and the characteristics of the match itself [2]. For this reason, the physical variables per minute, like relative distance, seem to be more representative of the general intensity of futsal and may be used as an overall index to provide more precise information about the demands of this sport [17].

Previous literature on team sport has demonstrated that physical performance is influenced by periods of a match and each player position has different physical demands [20,21]. Futsal studies have revealed no differences between playing positions [2,18]. However, dissimilar results have been shown related to game half comparisons [14,17,19]. These futsal requirements during elite official games have not been sufficiently widely studied as the accurate technology to analyze them does not currently exist. The investigation into these aspects can contribute to improved knowledge of futsal.

Therefore, the aim of this study was to analyze the influence of the match half and the playing position on physical requirements in the Spanish Professional Futsal League players during official games using a Local Positioning System.

## 2. Materials and Methods

### 2.1. Experimental Approach to the Problem

UWB technology system data were collected from 10 competitive matches from the Spanish Professional Futsal League (LNFS) during the 2019–2020 season. This enabled absolute and relative external training loads to be quantified for various playing positions and it was divided in first and second halves. Data were recorded from 05/10/2019 to 22/02/2020 in 10 consecutive home matches for the selected team. Matches were played at the local stadium every 15 days with 1 recovery week between the last match played and the recorded match.

### 2.2. Participants

A total of 14 elite futsal players (age 30.21 ± 3.98 years; height 1.77 ± 0.07 m; weight 74.85 ± 6.40 kg) from a professional club of Spanish Futsal League (LNFS) First Division participated in this study. Players who participated for at least 1 min in each game were included in the study. A total of 188 observations were recorded. All players were informed of the study requirements and provided written informed consent. The positions of the players were categorized into defenders (*n* = 3), wingers (*n* = 3) and pivots (*n* = 8) [18]. Players were included when they had participated in a considerable part of each match (minimum match time of 10 min) [5]. No goalkeepers were included. The power of the statistical results ranges from 0.86 to 0.99 for the selected sample. The study protocol was approved and followed the guidelines established by the local institution—the Ethics Committee of the European University of Madrid (CIPI35/2019)—and in accordance with the recommendations of the Declaration of Helsinki.

### 2.3. Equipment

The movement patterns of players during each match were monitored using WIMU PRO^TM^ LPS (RealTrack System SL, Almería, Spain) with UWB technology. This system is composed of two sub-systems: the reference system and WIMU PRO^TM^ inertial device (transported by the player). Each dispositive has its own internal microprocessor, with a high-speed USB interface, to record, store and upload data [10]. The dispositives are composed of different sensors (four accelerometers, a gyroscope, a magnetometer, a Global Navigation Satellite System (GNSS), and an UWB chipset with a frequency for the chip’s signal of 18 Hz [22,23]. The reference system is composed of six antennas that are transmitters and receivers of the radio frequency signal. The antennas (mainly the master antenna) perform the computerizing of the position of the devices that are in their area of performance, while the devices receive that calculation [10], the radio frequency signal is almost under the same principle as the GPS system [22,23]. The validity and accuracy of this type of LPS has been demonstrated exposed in reference to determining distance covered, speed, mean velocity, accelerations and decelerations for intermittent activities [10,11,13,24,25,26].

### 2.4. Procedures

**Figure 1:**
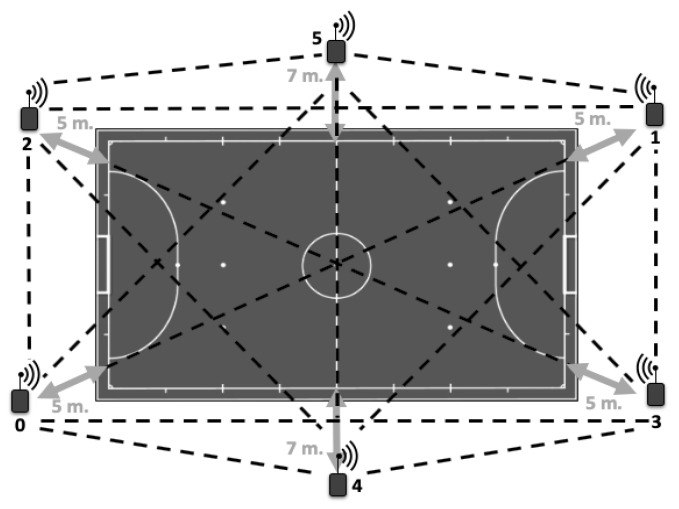
Antenna distribution of the Local Positioning System and distance reference to the futsal pitch. Arrows indicate distance from the antennas to the court and black lines indicate communication between antennas.

The Local Positioning System was installed on the futsal pitch where the team played their matches in concordance to user manual and previous studies [10,23]. The distribution of six antennas (Figure 1) with UWB technology were fixed 5 m from the perimeter line of the field, except for those located at the middle line of the field, which were fixed 7 m from the perimeter.

This way, the antennas formed a hexagon for better signal emission and reception. Once installed, they were switched on one-by-one, with the master antenna turned on last, and a process of autocalibration of the antennas was carried out for 5 min, where the master synchronized all antennas with a common clock. After, the tracking devices were switched on and a process of recognition and automatic communication with the antenna was carried out for 1 min. A researcher equipped with a device traversed the pitch lines to stablish the perimeter of the court and project it in the SPRO^TM^ software. Finally, one individual device was placed to the upper back of each player using a specially adjustable vest before commencing the match.

### 2.5. Data Processing

The physical activity variables were considered in accordance with previous futsal studies [4,19,27]. The specific software (SPRO^TM^ v. 958) was used to analyze and report on the performance data of the players for each match. The player time was calculated by specific software through the perimeter of the court considering the effective time that player played during each game. The external load variables examined were: Time Player (min); Total Distance Covered (m); Relative Distance (m·min^−1^); Explosive Distance (distance with ACC > 1.12 m·s^−2^), High-Intensity Break Distance (HIBD: distance with DEC > 2 m·s^−2^); High-Speed Running Distance (HSR: >15.1 km·h^−1^). Distance covered in speed zones, which were established by previous researchers in futsal [19]: ZONE 1: walking and low-intensity running (0‒10 km·h^−1^), ZONE 2: medium-intensity running (10.1‒15 km·h^−1^), ZONE 3: high-intensity running (>15.1 km·h^−1^), ZONE 4: sprinting (>18.1 km·h^−1^). Number of sprints (*n*·min^−1^); Maximal Speed (V_MAX:_ km·h^−1^); Mean Speed (V_MEAN:_ km·h^−1^). Number of Accelerations (*n*·min^−1^) and Decelerations (*n*·min^−1^) per minute; Maximal Acceleration (ACC_MAX:_ m·s^−2^) and Deceleration (DEC_MAX:_ m·s^−2^); Mean Acceleration (ACC_MEAN:_ m·s^−2^) and Deceleration (DEC_MEAN:_ m·s^−2^); number of accelerations and decelerations by zones: ZONE 1: low (2‒3 m·s^−2^); ZONE 2: medium (3‒4 m·s^−2^); ZONE 3: high (4‒5 m·s^−2^); ZONE 4: very high (5‒6 m·s^−2^).

### 2.6. Statistical Analysis

Data are presented as means ± standard deviations. The level of significance was set at *p* < 0.05. Before carrying out the analyses, the Kolmogorov‒Smirnov distribution test was performed to confirm a normal distribution of the variables. Differences between groups were evaluated through mixed two-way ANOVA (first half vs. second half as repeated measure, and defender vs. pivot vs. winger as independent measure). The post hoc analysis was adjusted using the Bonferroni method. Furthermore, two different effect sizes were calculated. For group effects, partial Eta-squared (*ηp^2^*) was calculated with the following interpretation: small (*ηp^2^* = 0.01–0.059); medium (*ηp^2^* = 0.06−0.14); and large effect (*ηp^2^* > 0.14). For the post-hoc analysis, Cohen’s d (ES) was calculated and defined as follows: trivial (ES < 0.19); small (ES = 0.2–0.49); medium (ES = 0.50–0.79) and large (ES > 0.8). All data were statistically analyzed using SPSS V24.0 for Windows.

## 3. Results

The descriptive outcomes presented a player time of 37.10 ± 13.60 min and a total distance covered of 3375 ± 1139 m. When comparing overall differences across the three positions (defender, pivot and winger) we found a significant group effect for relative distance, explosive distance, HIBD, HSR, DEC_MAX_, number of sprints, distance covered in zone 2, zone 3 and zone 4 (*ηp*^2^ from 0.048 to 0.095, Table 1). Any significant group effects were found across two halves, not for the interaction effect (Table 1).

**Figure 2:**
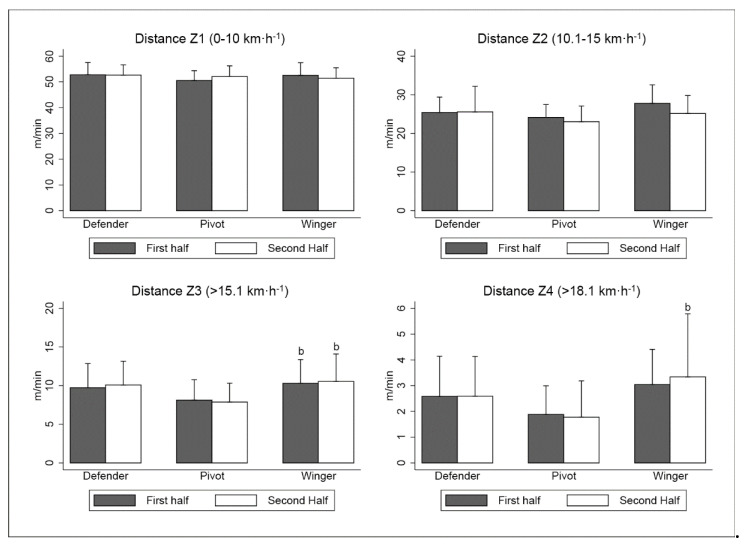
Relative distance covered in different speed ranges. ^b^ significant differences with respect to pivot (*p* < 0.05). Abbreviations: Z1, zone 1; Z2, zone 2; Z3, zone 3; Z4, zone 4.

With regard to the relative distance covered in different speed ranges, wingers evidenced a significant reduction in distance covered from zone 2 during the second half (−2.61 m·min^−1^; CI 95%: −4.42 to −0.80; ES: 0.55; Figure 2). The other player positions did not show any difference in distances covered between the two halves (*p* > 0.05; Figure 1). In terms of player positions, wingers covered a greater HRS distance than pivots throughout both the first half (+2.18 m·min^−1^; CI 95%: 0.01 to 4.35; ES: 0.76) and the second half (+2.67 m·min^−1^; CI 95%: 0.56 to 4.78; ES: 0.89). In addition, the sprint distance covered by wingers was larger than that by pivots during the second period (+1.56 m·min^−1^; CI 95%: 0.37 to 2.76; ES: 0.81).

**Figure 3:**
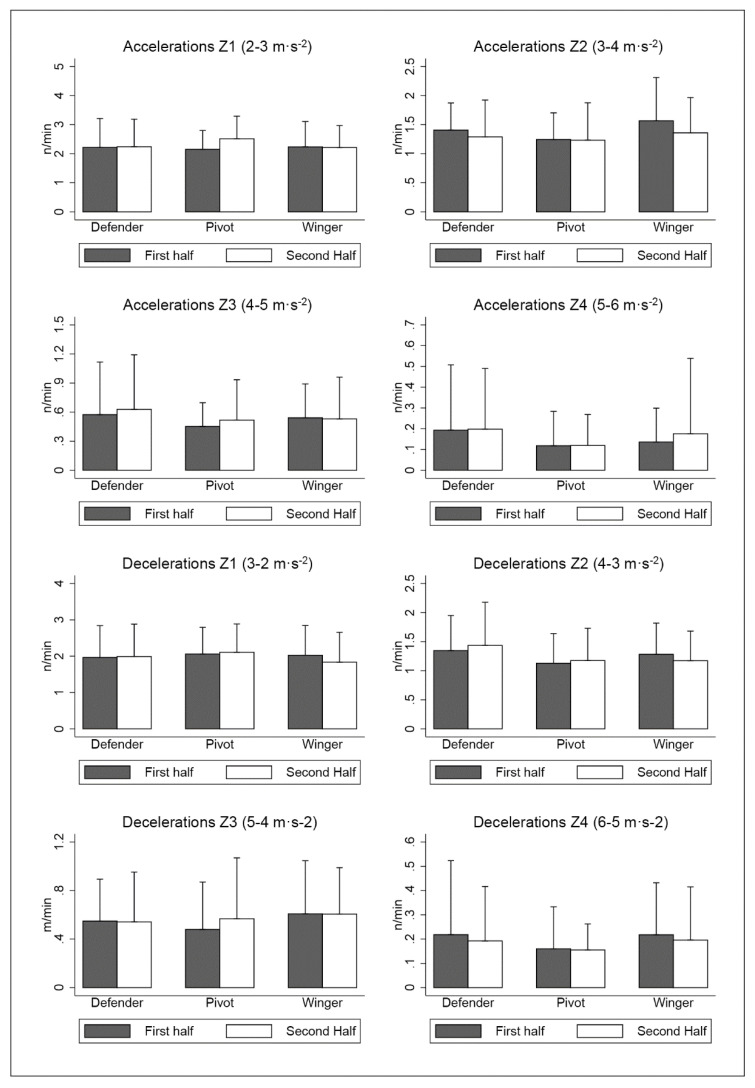
Number of acceleration and deceleration per minute in different speed ranges. Abbreviations: Z1, zone 1; Z2, zone 2; Z3, zone 3; Z4, zone 4.

The analysis of the accelerations and decelerations in different ranges of intensity did not show significant differences related to players’ position and the period of the match (*p* > 0.05; Figure 3).

## 4. Discussion

This is the first study to investigate the physical demands of elite futsal players during official matches from the First Division of the Spanish Professional Futsal League. For this purpose, a new technology, i.e., the Local Positioning System [9], was used to compare the physical demands between the first and the second half of matches and according to the playing position. The main findings were that most of the physical load executed by players did not change when comparing between the game periods. Moreover, this study revealed different physical demands according to the playing position.

Previous studies of futsal investigated the performance of professional players during official matches [14,18,19] or simulated games [4,27]. Additionally, some research analyzes the performance of futsal players with different playing levels [5,27,28,29]. However, these investigations used video-tracking analysis instead of a local positioning system. This difference in methodology might explain the lower distance covered per minute in this manuscript compared to previous studies [4,5,30]. Additionally, it is important to consider the competition-level differences from the research that have been evidenced to affect physical variables like distance covered per minute [31]. The data in this manuscript were obtained from official matches from the highest league in Spain and the level for each competition can have different physical demands. In fact, the outcomes from the Brazilian First Division [19], one of the most important leagues together with the Spanish one, reveal similar values to this variable. In contrast, Portuguese professional futsal league revealed greater findings than the present investigation in this variable [14]. It may be due to the differences in the format of competition examined in both studies.

As regards the total distance covered by players, the previous investigations showed ~25% greater distances than this study [5,17,27]. Simultaneously, a recent investigation on official futsal games [14] showed similar results that the present study. These findings demonstrate that this variable depended on the amount of time spent participating in the game, as has previously been reported [17]. Additionally, two studies relativized the distance covered per minute in different ranges [4,14] and evaluated only sprint actions [18]. Although there are differences in methodology between this study and the one presented in this manuscript, somehow both works show similar values. Most of the distance covered by the players is performed with low-intensity (51.99 ± 4.28 m·min^−1^) and medium-intensity (25.18 ± 4.56 m·min^−1^) running, while high-intensity running (9.44 ± 2.98 m·min^−1^) and sprint running (2.53 ± 1.57 m·min^−1^) represented much lower distances. These outcomes help in determining the specific physical performance profile of futsal players in reference to this variable.

The comparation of performance between halves of the match has been studied on football principally with evidence of significant diminution in most physical variables in the second half [32]. Some research in futsal is in line with this finding showing a significant decrease in relative distance covered and variations in total distance covered [17,19]. These outcomes are contrary to the ones presented in this manuscript, as a deterioration in the physical variables was not evidenced. These results coincide with current investigation of elite futsal players [14], which observed similar values between the first and second half. The differences between other studies might be due to the fact that distance works as a measure of volume [33]. Therefore, this variable is influenced by the overall duration of the game as well as the total time each player spends participating in the match. Since futsal is a sport with unlimited substitutions allowed, the total distance covered should not be taken as a performance indicator [14,17], as it is more appropriate to relativize this variable to minutes of play.

When comparing distances at different intensities or as a proportion of the high-intensity running, the previous studies did not reveal significant differences between halves [14,30]. Moreover, another study did not find differences in sprint distances between first and second halves either when comparing analyses of five official matches in the Brazilian First Division Futsal League [18]. In the present manuscript, the performance of high-intensity actions did not decrease significantly between halves either. These results suggest that these performances remain constant regardless of the game duration and the game period [18]. The main reason for these outcomes is the unlimited substitutions rule that avoids players accumulating high levels of fatigue that negatively affect their physical performance [18,30]. This suggests that futsal players are able to maintain a high level of performance anytime they participate in a match. Furthermore, it is important to bear in mind that futsal training is usually aimed at improving the ability of players to deal with repeated sprint actions, providing the players with a higher capacity to recover from these kinds of efforts [5], and thereby avoiding a reduction in performance during the games.

Furthermore, it was emphasized that variables such as high-speed running distance (zone 4) and sprinting distance (zone 5) were somewhat greater through the second half among defenders and wingers. This needs further investigation as it is possible that the requirements of the game or the amount of time players spend participating in these positions could have an influence, as has been observed in other team sports [20,34].

The fact that physical requirements vary according to the players’ position has been widely evidenced in other indoor team sports [21,35], but it has not been studied in futsal before now. This manuscript evidences some differences according to the players’ position, but these differences are much lower than those reported in football [20]. This is probably due to the existence of so many specific positions that do not exist in futsal, and all players are versatile so they might have different roles or positions during the games [18]. The main differences among players were found between pivots and wingers. These differences may be due to the technical and tactical requirements of each position in the offensive role [3]. Wingers usually play at a fast speed with continuous explosive action such as dribbling, and they move a lot around the pitch [36]. Pivots usually execute brief efforts and maintain a permanent position near the goal on the field. On the other hand, defenders and wingers show similar values with their physical actions. The absence of major differences between these two positions is probably because these players commonly exchange their position during matches as they perform different roles and functions in the game [18].

In terms of acceleration and deceleration actions, there is only one reference concerning futsal [14]. The results of the present study are greater in comparison with other professional futsal leagues [14]. These differences could be due to design of research, because of the prior study did not compare playing position. Futsal is considered a multidirectional sport in which agility is key for performance [6,37]. This investigation is in line with this idea as it is evidenced by the amount of accelerations and decelerations. In fact, we found a balance between the number of accelerations and decelerations like previous research on elite futsal players [14], which suggests that futsal is a sport that demands similar fast sprinting and braking. These actions are produced in many different axes that correspond to different skills such as sprinting or changing direction [21]. Therefore, executions of technical and tactical movements in relation to attacking or defensive actions may be factors that influence these types of demands [35]. Although there were no significant differences according to playing position, defenders presented greater acceleration (9.41 ± 9.73 *n*·min^−1^) and deceleration (9.12 ± 9.75 *n*·min^−1^) than other specific positions. One possible explanation for this might be that a player in this position depends greatly on the opposite player when performing a defensive role and then reacting quickly to that player’s actions. Therefore, players in this position need to respond continuously to attacking players’ actions [21].

The possible limitation of the current study might be that only one team was analyzed. For this reason, the results should be cautiously interpreted. However, the quality of the players can serve as a reference about the physical demands on futsal players. The influence on contextual variables such as defensive or attacking role, match results, opposition level, or goalkeeper–player situations in physical performance was not examined and further research will be necessary. Furthermore, the data obtained in official competitions might help to detect and understand the worst-case scenarios. Additionally, the investigation of acceleration and deceleration variables could be interesting to establish a futsal player’s profile of these demands.

The analysis of a game’s physical demands ought to be helpful to develop futsal-specific training programs. This knowledge of match requirements could be used to regulate the training load with the purpose of optimization and individualization of players’ performance by professional staff.

## 5. Conclusions

The principal finding of the current manuscript did not evidence differences in the physical performance of players between the first and second half. Therefore, the unlimited substitutions rule, could be important to maintain the performance during the match. The physical requirements varied among pivots and wingers regarding high-intensity actions. Consequently, individualized training will be necessary to develop the specific physical requirement for a given playing position. These outcomes add new contributions to the understanding of futsal’s physical demands.

## Figures and Tables

**Table 1:** Physical actions during the first and second half of the futsal match according to the playing position.

Halves	Variable	Defender (1)			Pivot (2)			Winger (3)			Position Group Effect		Halves Group Effect	
											*p*	*ηp^2^*	*p*	*ηp^2^*
**First** **Half**	Relative Distance (m·min^−1^)	91.39	±	9.41	85.58	±	6.41	94.69	±	9.66 ^b^	0.001	0.079	0.545	0.002
	Explosive Distance (m·min^−1^)	14.53	±	2.57	13.40	±	2.04	15.72	±	2.25 ^b^	0.001	0.074	0.631	0.001
	HIBD (m·min^−1^)	5.04	±	1.56	4.45	±	0.94	5.61	±	1.11 ^b^	0.001	0.070	0.833	0.000
	HSR (m·min^−1^)	15.44	±	5.10	12.99	±	4.37	17.03	±	4.86 ^b^	0.000	0.095	0.836	0.000
	Accelerations (*n*·min^−1^)	9.41	±	9.73	7.42	±	8.18	8.04	±	8.09	0.569	0.006	0.987	0.000
	Decelerations (*n*·min^−1^)	9.12	±	9.75	7.37	±	8.14	7.77	±	8.15	0.546	0.007	0.967	0.000
	ACC_MAX_ (m·s^−2^)	4.95	±	0.63	5.00	±	0.45	5.19	±	0.48	0.120	0.023	0.962	0.000
	DEC_MAX_ (m·s^−2^)	−5.25	±	0.63	−5.43	±	0.56	−5.70	±	0.59 ^a^	0.000	0.091	0.900	0.000
	ACC_MEAN_ (m·s^−2^)	2.46	±	0.69	2.61	±	0.68	2.63	±	0.66	0.312	0.013	0.904	0.000
	DEC_MEAN_ (m·s^−2^)	−2.53	±	0.73	−2.64	±	0.70	−2.72	±	0.70	0.192	0.018	0.801	0.000
	V_MAX_ (km·h^−1^)	20.60	±	0.80	20.14	±	0.98	21.03	±	0.83	0.128	0.022	0.610	0.001
	V_MEAN_ (km·h^−1^)	6.26	±	0.39	6.03	±	0.40	6.46	±	0.45 *	0.063	0.030	0.223	0.008
	Number of sprints (*n*·min^−1^)	0.74	±	0.33	0.59	±	0.26	0.81	±	0.24	0.000	0.081	0.739	0.001
**Second Half**	Relative Distance (m·min^−1^)	91.80	±	12.00	85.58	±	9.01	91.50	±	9.39				
	Explosive Distance (m·min^−1^)	14.67	±	3.30	13.44	±	2.13	14.94	±	2.73				
	HIBD (m·min^−1^)	5.17	±	1.61	4.46	±	1.14	5.32	±	1.59				
	HSR (m·min^−1^)	16.17	±	5.43	12.30	±	3.98	17.54	±	6.35				
	Accelerations (*n*·min^−1^)	9.05	±	9.42	8.63	±	9.07	7.26	±	7.91				
	Decelerations (*n*·min^−1^)	8.87	±	9.42	8.28	±	9.14	6.94	±	7.87				
	ACC_MAX_ (m·s^−2^)	5.00	±	0.59	5.04	±	0.46	5.12	±	0.57				
	DEC_MAX_ (m·s^−2^)	−5.29	±	0.69	−5.41	±	0.57	−5.71	±	0.62				
	ACC_MEAN_ (m·s^−2^)	2.50	±	0.68	2.50	±	0.66	2.67	±	0.59				
	DEC_MEAN_ (m·s^−2^)	−2.56	±	0.70	−2.62	±	0.72	−2.80	±	0.65				
	V_MAX_ (km·h^−1^)	20.46	±	0.96	20.18	±	1.02	20.68	±	2.96				
	V_MEAN_ (km·h^−1^)	6.24	±	0.58	5.95	±	0.51	6.15	±	1.00				
	Number of sprints (*n*·min^−1^)	0.73	±	0.27	0.58	±	0.21	0.88	±	0.46 ^b^				

* Significant differences between the first and the second half (*p* < 0.05); ^a^ significant differences with respect to defender (*p* < 0.05), ^b^ significant differences with respect to pivot (*p* < 0.05). Abbreviations: *ηp*^2^, partial Eta-squared; HIBD, High Intensity Break Distance with DEC > 2 m·s*^−^*^2^; HSR, High Speed Running Distance > 15.1 km·h*^−^*^1^; ACC_MAX_, Maximal Acceleration: DEC_MAX_, Maximal Deceleration; ACC_MEAN_, Mean Acceleration; DEC_MEAN_, Mean Deceleration; V_max_, Maximal speed; V_MEAN_, Mean Speed.

## References

[1] Beato M., Coratella G., Schena F. (2016). Brief Review of the State of Art in Futsal. J. Sports Med. Phys. Fit..

[2] Naser N., Ali A., Macadam P. (2017). Physical and Physiological Demands of Futsal. J. Exerc. Sci. Fit..

[3] Méndez C., Gonçalves B., Santos J., Ribeiro J.N., Travassos B. (2019). Attacking Profiles of the Best Ranked Teams from Elite Futsal Leagues. Front. Psychol..

[4] Castagna C., D’Ottavio S., Vera J.G., Álvarez J.C.B. (2009). Match Demands of Professional Futsal: A Case Study. J. Sci. Med. Sport.

[5] Dogramaci S.N., Watsford M.L., Murphy A.J. (2011). Time-Motion Analysis of International and National Level Futsal. J. Strength Cond. Res..

[6] Taylor J.B., Wright A.A., Dischiavi S.L., Townsend M.A., Marmon A.R. (2017). Activity Demands During Multi-Directional Team Sports: A Systematic Review. Sport. Med..

[7] Chambers R., Gabbett T.J., Cole M.H., Beard A. (2015). The Use of Wearable Microsensors to Quantify Sport-Specific Movements. Sport. Med..

[8] Scott M.T.U., Scott T.J., Kelly V.G. (2016). The Validity and Reliability of Global Positioning Systems in Team Sport. J. Strength Cond. Res..

[9] Rico-González M., Los Arcos A., Rojas-Valverde D., Clemente F.M., Pino-Ortega J. (2020). A Survey to Assess the Quality of the Data Obtained by Radio-Frequency Technologies and Microelectromechanical Systems to Measure External Workload and Collective Behavior Variables in Team Sports. Sensors.

[10] Bastida-Castillo A., Gómez-Carmona C., De la Cruz-Sánchez E., Reche-Royo X., Ibáñez S., Pino Ortega J. (2019). Accuracy and Inter-Unit Reliability of Ultra-Wide-Band Tracking System in Indoor Exercise. Appl. Sci..

[11] Serpiello F.R., Hopkins W.G., Barnes S., Tavrou J., Duthie G.M., Aughey R.J., Ball K. (2018). Validity of an Ultra-Wideband Local Positioning System to Measure Locomotion in Indoor Sports. J. Sports Sci..

[12] Vazquez-Guerrero J., Reche X., Cos F., Casamichana D., Sampaio J. (2018). Changes in External Load When Modifying Rules of 5-on-5 Scrimmage Situations in Elite Basketball. J. Strength Cond. Res..

[13] Fleureau A., Lacome M., Buchheit M., Couturier A., Rabita G. (2020). Validity of an Ultra-Wideband Local Positioning System to Assess Specific Movements in Handball. Biol. Sport.

[14] Ribeiro J.N., Gonçalves B., Coutinho D., Brito J., Sampaio J., Travassos B. (2020). Activity Profile and Physical Performance of Match Play in Elite Futsal Players. Front. Psychol..

[15] Hirokawa R., Ebinuma T. (2009). A Low-Cost Tightly Coupled GPS/INS for Small UAVs Augmented with Multiple GPS Antennas. Navigation.

[16] Castagna C., Barbero Álvarez J.C. (2010). Physiological Demands of an Intermittent Futsal-Oriented High-Intensity Test. J. Strength Cond. Res..

[17] Barbero-Alvarez J.C., Soto V.M., Barbero-Alvarez V., Granda-Vera J. (2008). Match Analysis and Heart Rate of Futsal Players during Competition. J. Sports Sci..

[18] Caetano F.G., de Oliveira M.J., Marche A.L., Nakamura F.Y., Cunha S.A., Moura F.A. (2015). Characterization of the Sprint and Repeated-Sprint Sequences Performed by Professional Futsal Players, According to Playing Position, During Official Matches. J. Appl. Biomech..

[19] De Oliveira Bueno M.J., Caetano F.G., Pereira T.J.C., De Souza N.M., Moreira G.D., Nakamura F.Y., Cunha S.A., Moura F.A. (2014). Analysis of the Distance Covered by Brazilian Professional Futsal Players during Official Matches. Sport Biomech..

[20] Sarmento H., Marcelino R., Anguera M.T., Campaniço J., Matos N., Leitão J.C. (2014). Match Analysis in Football: A Systematic Review. J. Sports Sci..

[21] Vázquez-Guerrero J., Suarez-Arrones L., Casamichana Gómez D., Rodas G. (2018). Comparing External Total Load, Acceleration and Deceleration Outputs in Elite Basketball Players across Positions during Match Play. Kinesiology.

[22] Sczyslo S., Schroeder J., Galler S., Kaiser T. Hybrid Localization Using UWB and Inertial Sensors. Proceedings of the 2008 IEEE International Conference on Ultra-Wideband.

[23] García-Santos D., Pino-Ortega J., García-Rubio J., Vaquera A., Ibáñez S.J. (2019). Internal and External Demands in Basketball Referees during the U-16 European Women’s Championship. Int. J. Environ. Res. Public Health.

[24] Bastida Castillo A., Gómez Carmona C.D., Pino Ortega J., de la Cruz Sánchez E. (2017). Validity of an Inertial System to Measure Sprint Time and Sport Task Time: A Proposal for the Integration of Photocells in an Inertial System. Int. J. Perform. Anal. Sport.

[25] Stevens T.G.A., de Ruiter C.J., van Niel C., van de Rhee R., Beek P.J., Savelsbergh G.J.P. (2014). Measuring Acceleration and Deceleration in Soccer-Specific Movements Using a Local Position Measurement (LPM) System. Int. J. Sports Physiol. Perform..

[26] Gómez-Carmona C.D., Bastida-Castillo A., García-Rubio J., Ibáñez S.J., Pino-Ortega J. (2019). Static and Dynamic Reliability of WIMU PRO^TM^ Accelerometers According to Anatomical Placement. Proc. Inst. Mech. Eng. Part P J. Sport Eng. Technol..

[27] Makaje N., Ruangthai R., Arkarapanthu A., Yoopat P. (2012). Physiological Demands and Activity Profiles during Futsal Match Play According to Competitive Level. J. Sports Med. Phys. Fit..

[28] Álvarez J.C.B., D’ottavio S., Vera J.G., Castagna C. (2009). Aerobic Fitness in Futsal Players of Different Competitive Level. J. Strength Cond. Res..

[29] Naser N., Ali A. (2016). A Descriptive-Comparative Study of Performance Characteristics in Futsal Players of Different Levels. J. Sports Sci..

[30] Barbero Álvarez J.C. (2008). Heart Rate and Activity Profile for Young Female Soccer Players. J. Hum. Sport Exerc..

[31] Morgans R., Adams D., Mullen R., Sacramento J., McLellan C., Williams M. (2015). A Comparison of Physical and Technical Match Performance of a Team Competing in the English Championship League and Then the English Premier League Following Promotion. Int. J. Sports Sci. Coach..

[32] Castellano J., Blanco-Villaseñor A., Álvarez D. (2011). Contextual Variables and Time-Motion Analysis in Soccer. Int. J. Sports Med..

[33] Rago V., Brito J., Figueiredo P., Costa J., Barreira D., Krustrup P., Rebelo A. (2020). Methods to Collect and Interpret External Training Load Using Microtechnology Incorporating GPS in Professional Football: A Systematic Review. Res. Sport Med..

[34] Stojanović E., Stojiljković N., Scanlan A.T., Dalbo V.J., Berkelmans D.M., Milanović Z. (2018). The Activity Demands and Physiological Responses Encountered During Basketball Match-Play: A Systematic Review. Sport Med..

[35] Kempton T., Sirotic A.C., Coutts A.J. (2017). A Comparison of Physical and Technical Performance Profiles Between Successful and Less-Successful Professional Rugby League Teams. Int. J. Sports Physiol. Perform..

[36] López J.V. (2017). UEFA Futsal Coaching Manual.

[37] Milanović Z., Sporiš G., Trajković N., Fiorentini F. (2011). Differences in Agility Performance between Futsal and Soccer Players. Sport Sci..

